# Type 2 Diabetes Mellitus and Hearing Loss: A Prisma Systematic Review and Meta‐Analysis

**DOI:** 10.1002/ohn.1346

**Published:** 2025-09-08

**Authors:** Miguel Caballero‐Borrego, Ivan Andujar‐Lara

**Affiliations:** ^1^ Department of Otorhinolaryngology Hospital Clínic Barcelona Spain; ^2^ Departament de Cirurgia i Especialitats Medicoquirúrgiques, Facultat de Medicina i Ciències de la Salut Universitat de Barcelona Barcelona Spain; ^3^ Systemic Autoimmune Diseases research group Institut d'Investigació Biomèdica August Pi Sunyer (IDIBAPS) Barcelona Spain

**Keywords:** hearing loss, diabetes mellitus, inner ear, hearing impairment, risk factor, deafness

## Abstract

**Objective:**

To conduct a systematic review and meta‐analysis to assess the association between type 2 diabetes and hearing loss.

**Data Sources:**

Search conducted in PubMed and Scopus databases for articles published between January 2019 and April 2024.

**Review Methods:**

Quality assessment and risk of bias analysis were conducted using the Newcastle‐Ottawa scale, and meta‐analyses of pooled data were performed with Cochrane's Review Manager.

**Results:**

From 8354 initially identified articles, 17 met the inclusion criteria. The prevalence of hearing loss in patients with type 2 diabetes range from 40.6% to 71.9%, which was significantly higher than in controls (odds ratio [OR]: 4.19; 95% confidence interval [CI]: 1.22‐14.37). The mean pure‐tone audiometric thresholds of the diabetic group were 3.19 dB higher (95% CI: 1.08‐5.19) and were higher for both low frequencies (1.11 dB, 95% CI: 0.62‐1.57) and, more severely, for high frequencies (2.3 dB, 95% CI: 1.97‐2.63). Patients with moderate (0.57%, 95% CI: 0.1‐1.05) and severe‐to‐profound (0.95%, 95% CI: 0.02‐1.87) hearing loss had higher mean HbA1c levels than controls. The prevalence of hearing loss was significantly higher among patients with a diagnosis of diabetes for more than 10 years (OR 2.07, 95% CI: 1.45‐2.94). The gender of diabetic patients was found to have no influence.

**Conclusion:**

The prevalence of hearing loss among individuals with type 2 diabetes ranges from 40.6% to 71.9%. Compared to the control group, the risk of hearing loss is 4.19 times higher and is predominantly observed at higher frequencies. The duration of diabetes and HbA1c levels are significant factors in the prevalence of hearing loss.

Diabetes mellitus (DM) is a chronic metabolic disease characterized by persistent hyperglycemia and impaired protein, carbohydrate and fat metabolism, due to insulin dysregulation. According to the World Health Organization, the number of people with diabetes increased from 108 million in 1980 to 422 million in 2014. Notably, this increase has been more pronounced in low‐ and middle‐income countries than in high‐income countries.^1^

There are 2 main types of diabetes: type 1 diabetes mellitus (T1DM) and type 2 diabetes mellitus (T2DM).^2^ T1DM is a metabolic disorder that results from the autoimmune destruction of the pancreatic beta (β) cells, leading to a reduction or complete absence of insulin production. It accounts for 5.0% to 10.0% of all DM cases and can manifest at any age, although its incidence typically reaches its peaks between birth and 14 years of age in most populations.^3^ This condition is chronic and progressive throughout the lifespan. In contrast, T2DM, which accounts for 90.0% of all cases of DM, is characterized by a progressive decline in insulin secretion on a background of insulin resistance.^3^, ^4^, ^5^

Persistent hyperglycemia has been demonstrated to result in damage to both microvascular and macrovascular structures, which represents the underlying pathophysiology of the complications associated with DM.^6^, ^7^ It has been estimated by some authors that more than 72.0% of patients will experience at least 1 complication. These may be microvascular in nature, although in up to 1 in 4 patients, both types may be experienced.^8^ Such complications typically encompass retinopathy, nephropathy, peripheral neuropathy, and cardiovascular damage.^9^, ^10^

As a systemic disease, there is a possibility that it may affect the auditory system.^11^ The physiology of the cochlea is indirectly affected in T2DM because of microcirculatory alterations, resulting in changes to the ultrastructure of the inner ear capillaries. These changes include thickening of the basilar membrane and atrophy of the stria vascularis.^3^, ^12^ There is growing evidence of an association between T2DM and hearing loss (HL). This has been demonstrated by studies comparing the prevalence of HL in adults with diabetes with that in healthy controls.^8^ However, clinical studies have not consistently demonstrated a positive association.^13^ Age and duration of diabetes have also been proposed as risk factors for HL in diabetic patients.^14^

The objective of this study is to perform a systematic review and meta‐analysis to evaluate the association between T2DM and HL. The objective is to estimate the prevalence of hearing impairment among patients with diabetes, examine the mean difference in hearing thresholds between T2DM patients and controls, and investigate how factors such as duration of diabetes, HbA1c levels and gender may affect hearing loss in those diagnosed with this condition.

## Methods

The systematic review is structured in accordance with the PRISMA 2020 statement/checklist in terms of its structure and format. The research question was formulated in accordance with the PICO (Population, Intervention, Comparison, Outcome) framework. The population (P) is constituted by individuals diagnosed with T2DM, the intervention (I) is audiometric testing, the comparison (C) group is composed of healthy subjects, and the outcome (O) is hearing loss. Therefore, the question is: “Are type 2 diabetic patients at a higher risk of hearing loss, as measured by audiometric tests, in comparison to healthy controls?”

A protocol for this systematic review and meta‐analysis was designed and subsequently registered in the PROSPERO database prior to the commencement of study (registration number: CRD42024539100).

### Eligibility Criteria

This systematic review included cohort, case‐control, and cross‐sectional studies that investigated the association between DM and HL. Inclusion criteria required a comprehensive elucidation of hearing impairment with results requiring precise measurement through audiometric tests, and diabetes, diagnosed through medical record or through the American Diabetes Association (ADA) criteria for the diagnosis of diabetes: (1) HbA1c ≥ 6.5% or (2) free plasma glucose ≥126 mg/dL or (3) 2‐hour plasma glucose ≥200 mg/dL during an oral glucose tolerance test or (4) random glucose test ≥200 mg/dL with classic symptoms of hyperglycemia or hyperglycemic crisis.^15^ The review encompassed studies involving individuals of all ages, genders, races, and national backgrounds. Exclusion criteria comprised studies that failed to exclude alternative causes for hearing impairment, those involving only T1DM, those addressing sudden hearing loss, those with unreliable audiometric tests, and those involving non‐human subjects. Any type of publication other than observational studies was excluded. Furthermore, the review was limited to studies published within the timeframe of January 1, 2019, to April 1, 2024, inclusive, and available in either English or Spanish.

### Information Sources

The investigation was conducted using the PubMed and Scopus databases. The search methodology employed the use of keywords and Medical Subject Headings (MeSH) terms associated with diabetes, hearing loss, and the confluence of both disorders. Moreover, grey literature was not included in the search.

### Search Strategy

The search strategy included the following terms and keywords: (“diabetes” OR “diabetes mellitus” OR “type 2 diabetes mellitus” OR “diabetic”) AND (“hearing loss” OR “hearing impairment” OR “auditory”). The references extracted from the retrieved articles were subjected to a comprehensive examination to identify any pertinent studies that may have been overlooked during the review process.

### Selection Process

The preliminary stage of the review process entailed the formulation of eligibility criteria by the 2 investigators. They subsequently conducted independent evaluations of the titles and abstracts derived from the electronic search, with the objective of compiling a list of relevant articles. Subsequently, a collaborative assessment was conducted to generate a unified list. All citations deemed relevant for the second‐stage review were subjected to a comprehensive examination to determine their inclusion or exclusion. This evaluation was initially conducted independently and subsequently through a collaborative process involving the authors. Any discrepancies among reviewers were resolved through consensus.

### Data Collection Process

The 2 investigators conducted data extraction independently, employing a standardized data‐gathering form. In cases where the eligibility of the abstract was unclear, the complete article was retrieved for further clarification. Any issues encountered were resolved through discussion. The gathered data from the qualifying studies encompassed various aspects, including details pertaining to the studies themselves (such as author, publication year, and study design), the demographic characteristics of the patients (including the number, mean age values, and duration of diabetes), the methods employed for auditory evaluation, the prevalence of hearing loss, the key findings, and any relevant statistical analyses. Automation tools were not used during the data collection process.

### Data Items

This review focuses on studies that examine hearing loss. Any assessment of hearing loss was deemed eligible for inclusion, provided that the tests or diagnostic criteria employed exhibited sufficient validity and reliability. Studies failing to meet these criteria were excluded. It was expected that many different methods of assessing hearing loss would be used across studies. The following tests were assessed: (a) pure‐tone audiometry test, (b) speech test, (c) auditory brainstem response, (d) tympanometry test and (e) otoacoustic emissions test. For the purposes of analysis, PTA was selected due to its widespread availability. The hearing loss thresholds utilized in this article are classified as mild hearing loss (26‐40 dB), moderate hearing loss (41‐55 dB), and severe to profound hearing loss (greater than 56 dB).

We systematically collected information on the characteristics of the included studies and their results, focusing on 2 key areas: (1) study identifiers such as references, location, start date, and study design; and (2) participant characteristics, including age, gender, comorbidities and any other pre‐established eligibility criteria.

### Risk of Bias Assessment

Eligible articles underwent a quality assessment using the Newcastle‐Ottawa Quality Assessment Tool. This scale assesses 3 quality parameters (selection, comparability, and outcome) divided into 9 specific items. The articles were then categorized into 3 quality levels: high quality, moderate quality, and low quality. Only articles that scored at least 5 out of 9 on this scale, with at least 2 points for each parameter (1 point considered sufficient for comparability), were considered for inclusion in the final review. The selected articles were independently assessed by 2 authors in a blinded fashion, with subsequent comparison to determine inter‐rater reliability.

### Effect Measures

Our analysis strategy included the calculation of odds ratios (OR) to assess dichotomous outcomes, such as the influence of gender, diabetes duration, or diabetes prevalence. To quantify the magnitude of HL and HbA1c levels on HL, continuous variables, we used the standardized mean difference along with its corresponding 95.0% confidence interval.

### Synthesis Methods

Due to the complexity of the syntheses, studies with clearly defined criteria and diagnoses of HL in people with diabetes were included in the review to examine prevalence. Severity of the HL was assessed by reviewing studies that provided precise measurements of hearing thresholds by PTA. For the influence of gender and disease duration, studies with clear demographic information about the population studied were analyzed.

To include the collected data from the studies in our review, it was necessary to combine the standard deviations (SD) using Cochrane's algorithm for SD combination. Furthermore, in 1 study, Theodoroff et al, a normal distribution of the data was assumed in order to calculate SD from a mean, maximum value, and minimum value.^16^ This assumption was based on the understanding that 68.0% of the data falls within 1 standard deviation from the mean, 95.0% falls within 2 standard deviations and 99.7% falls within 3 standard deviations from the mean.

The utilization of tables facilitated the systematic examination of the characteristics and principal outcomes of the studies in question. The forest plot was the principal graphical instrument employed in the execution of a meta‐analysis. This visual representation of the data provides a comprehensive overview of the effect estimates and confidence intervals of individual studies, along with the summary estimate.

The meta‐analysis was conducted using the Cochrane Review Manager Software (Review Manager 5.3). The data pertaining to for prevalence, influence, and disease duration were entered in a dichotomous format. The data pertaining to PTA or HbA1c levels were entered in a continuous format. In the case of the former, the pooled odds ratio was obtained using a Mantel‐Haenszel effect mode. The presence of heterogeneity was assessed using a chi‐square test and the *I*² statistic, which provides an estimate of the total variation across studies. For continuous variables using PTA thresholds, an inverse variance and random or fixed effect analysis model was used.

### Reporting Bias Assessment

To assess selective reporting bias, a comparison was made between the planned measurements and outcomes described by the original trial investigators and those documented in the published paper. This involved cross‐referencing trial protocols (when available) with the content presented in the final publication. In cases where trial protocols were not publicly available, we analyzed the methods and results sections of the published papers. Additionally, we used our clinical expertise to identify cases where investigators did not report commonly used outcome measures.

### Certainty Assessment

The 5 GRADE criteria (study limitations, consistency of effect, imprecision, indirectness, and publication bias) were employed to assess the level of certainty of the evidence base concerning the studies that provided data for this study. The certainty of the evidence was classified as: (I) high, (II) moderate, (III) low, or (IV) very low.

## Results

A total of 8354 articles were identified through the initial search. An additional 2 articles were identified through a search of the references of selected articles. Following an independent and collaborative evaluation of the titles and abstracts, 87 articles were selected for a full review. However, 7 of the articles could not be accessed in full. Of the remaining 80 articles, 56 articles were subsequently excluded (1, 2). The remaining 30 articles were subjected to a quality assessment using the Ottawa‐Newcastle scale. A total of 17 articles were identified as meeting the requisite standards and were therefore included for detailed data extraction and further analysis.^16^, ^17^, ^18^, ^19^, ^20^, ^21^, ^22^, ^23^, ^24^, ^25^, ^26^, ^27^, ^28^, ^29^, ^30^, ^31^, ^32^ Agreement between raters was achieved through consensus, in accordance with the predefined criteria.

**Figure 1:**
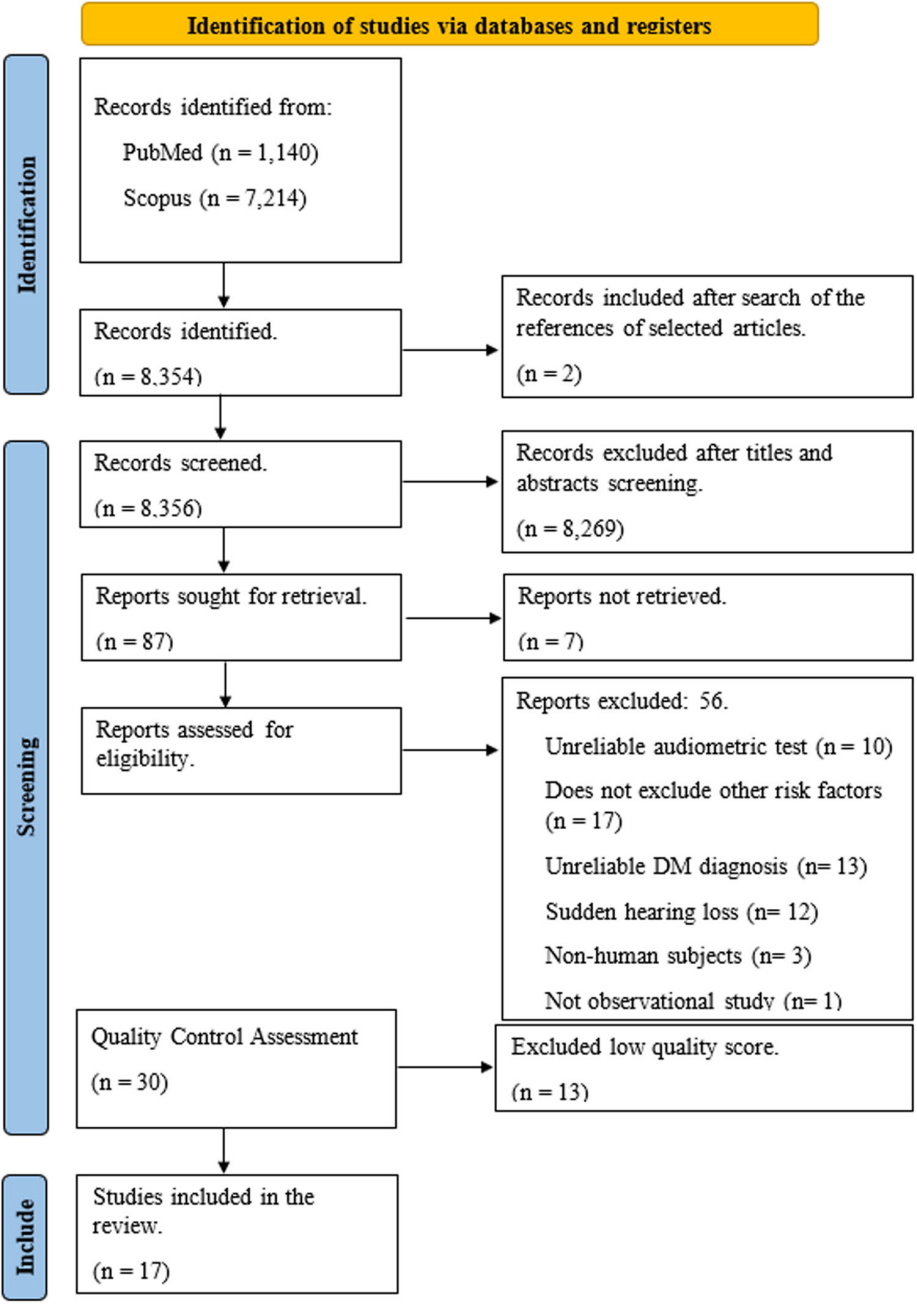
Flow diagram for study selection of articles looking at the effects of type 2 diabetes on hearing loss.

The analysis encompassed a total of 3910 individuals diagnosed with diabetes and 4,084 control subjects. Table 1 presents a comprehensive overview of the basic information and distinct characteristics of the recruited studies.

**Table 1:** Characteristics of the Included Studies

Author (year)	Country	Leve of evidence (GRADE)	NOS	Participants (diabetic/control)	Mean age (years)	Method of hearing assessment	Mean PTA thresholds (dB) (diabetic/control)
Lee HJ (2023)^29^	South Korea	III	High Quality	2039/3248	70.4/64.4	PTA	31.5/27.7
Madhan S (2023)^32^	India	III	Moderate Quality	32/32	44.03/45.56	PTA	32.66/22.24
Alizadeh Y (2022)^30^	Iran	III	High Quality	315/105	59.8/60.0	PTA	NA
Theodoroff SM (2021)^16^	USA	III	High Quality	97/93	45.9/49.0	PTA	18.41/16.16
Nourizadeh N (2021)^26^	Iran	III	High Quality	25/25	51.32/51.12	PTA	18.8/14.61
Vergou Z (2019)^27^	Greece	III	High Quality	227/221	60.9/56.5	PTA/DPOAEs	NA
Li Y (2020)^25^	China	III	High Quality	65/60	45.8/45.1	PTA/HFA/DPOAEs	NA
Goyal I (2019)^24^	India	III	High Quality	50/50	48.24/45.54	PTA/ABR	NA
Abou‐Elew M (2022)^31^	Egypt	III	High Quality	60/60	51.23/49.98	PTA, SRT	NA
Mishra IS (2023)^23^	India	III	High Quality	30/30	38.16/38.26	PTA/BAEP	NA
Elhussieny FM (2023)^19^	Egypt	III	High Quality	84/32	54.35/54.17	PTA	NA
Cho WK (2020)^28^	South Korea	III	High Quality	101/128	55.10/54.00	PTA, DPOAEs, ABR	12.26/11.14
*NO CONTROLS*							
Mishra UP (2024)^23^	Saudi Arabia	III	High Quality	152/NA	49.0/NA	PTA	NA
Al‐Abed SA (2023)^18^	Jordan	III	High Quality	118/NA	60.6/NA	PTA	NA
Shafiepour M (2022)^20^	Iran	III	High Quality	94/NA	61.54/NA	PTA	NA
Pillay S (2021)^22^	South Africa	III	High Quality	264/NA	59.0/NA	PTA	NA
Al‐Rubeaan K (2021)^21^	Saudi Arabia	III	High Quality	157/NA	51.0/NA	PTA/DPOAEs	NA

Abbreviations: ABR, auditory brainstem evoked responses; BAEP, brainstem auditory evoked potential; DPOAEs, Distortion Product Otoacoustic Emissions; HFA, High‐frequency Audiometry; NA, not applicable; NOS, Newcastle‐Ottawa scale; PTA, pure tone audiometry; SRT, speech reception threshold.

### Prevalence of Hearing Loss

In order to ascertain the prevalence of HL among individuals with diabetes, 9 studies were utilized.^17^, ^22^, ^29^, ^31^, ^32^ The results were presented as a percentage of the total population sampled. Across these studies, the prevalence of HL among diabetic subjects was found to vary considerably, spanning from 40.6% to 71.9% (Table 2). The criteria for defining HL were generally consistent, with a pure tone average equal to or exceeding 25 dB at selected frequencies in the worse ear. However, Al‐Abed et al, adopted a different threshold, defining HL as a pure tone average equal to or exceeding 20 dB.^18^

**Table 2:** Relationship Between Mean Age of Diabetics, Diabetes Duration and Hearing Loss

Author (year)	Diabetics mean age (years)	Diabetes duration (years)	Diabetics prevalence of HL (%)
Al‐Abed SA (2023)^18^	60.6	13.9	71.9
Mishra UP (2024)^23^	49.9	NS	70.3
Pillay S (2021)^22^	59.0	9.0	56.4
Lee HJ (2023)^29^	70.4	NS	55.8
Shafiepour M (2022)^20^	61.54	NS	71.3
Al‐Rubeaan K (2021)^21^	51.0	12.9	66.2
Elhussieny FM (2023)^19^	54.35	11.6	42.9
Madhan S (2023)^32^	44.03	3.96	40.62
Abou‐Elew M (2022)^31^	51.23	NS	65

Abbreviations: HL, hearing loss; NS, not specified.

As shown in 1, 2, a total of 2358 individuals with diabetes and 3561 controls from 4 studies^27^, ^29^, ^31^, ^32^ were pooled to investigate the association between diabetes and the prevalence of HL. The OR indicated that the prevalence was 4.19 (95% CI: 1.22‐14.37) times higher in the type 2 DM patients than in the controls. A *Z*‐score of 2.28 yielded a *P*‐value of .02, and a heterogeneity score (*I*²) of 83.0% was observed.

**Figure 2:**
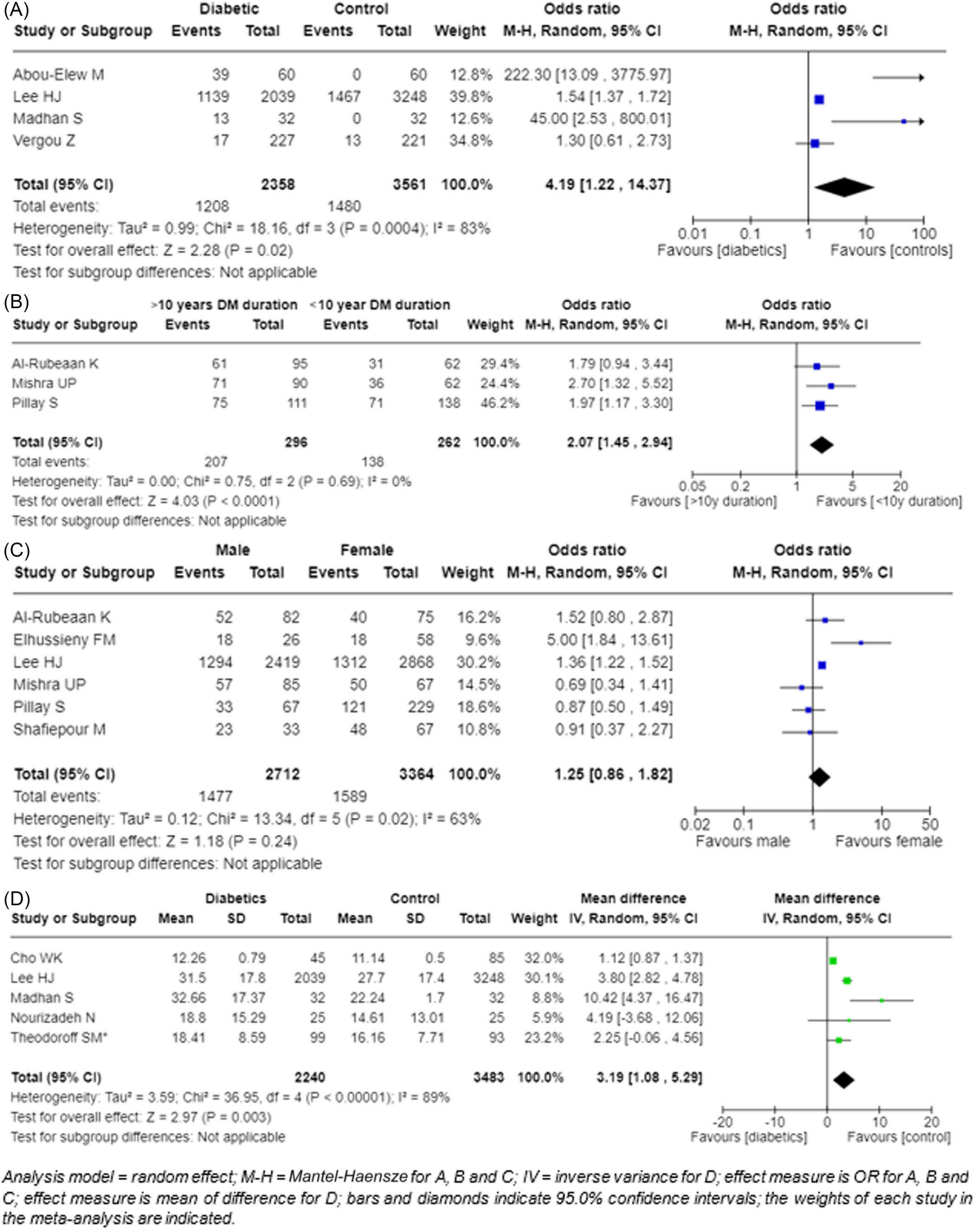
The prevalence of hearing loss among: (A) diabetics and controls; (B) duration of more or less than 10 years; (C) male and female subjects; and (D) mean audiometric thresholds in diabetics and controls.

### Duration of Diabetes and Prevalence of Hearing Loss

Three studies provided comprehensive information regarding the duration of diabetes and its association with the risk of HL.^17^, ^21^, ^22^ 1, 2 synthesized data pertaining to 558 diabetic patients. Of these, 296 had a diabetes duration exceeding 10 years, while 262 had a diabetes duration of less than 10 years. The OR demonstrated a 2.07 times higher risk of HL in patients with a diabetes duration of 10 years or more, with a 95.0% CI ranging from 1.45 to 2.94. The resulting *Z*‐score of 4.03 (*P* < .0001) evidences a significant association. The heterogeneity statistic (*I* ^2^) recorded a value of 0.0%, indicating a lack of heterogeneity across the studies included, which enhances the robustness of the findings.

Five studies offered thorough information regarding both the duration of diabetes and the mean age of the patients under study.^18^, ^19^, ^20^, ^21^, ^22^, ^32^ Al‐Abed et al^18^ recorded the highest mean age of patients (60.6 years old), reflecting an older cohort within the study population. This study also reported the highest prevalence of HL (71.9%) (Table 2). Conversely, Madhan et al, reported the youngest average age of patients (44.03 years old) and the lowest prevalence of HL among all the analyzed studies.^32^

### Gender and Prevalence of Hearing Loss

1, 2 illustrates that the analysis of 2712 diabetic males and 3364 diabetic females revealed no statistically significant differences between male and female patients diagnosed with diabetes in terms of the risk of HL.^19^, ^20^, ^21^, ^22^, ^23^, ^29^ The OR was calculated to be 1.25 (95% CI: 0.86‐1.82) in favor of male subjects. The *Z*‐score was determined to be 1.18 (*P* = .24), which suggests that no significant difference exits. Additionally, the *I* ^2^ was determined to be 63.0%, indicating a moderate variability across the studies.

### Pure‐Tone Audiometry Threshold

The mean PTA thresholds at 250, 500, 1000, 2000, 4000, and 8000 Hz were provided by 5 studies.^16^, ^26^, ^28^, ^29^, ^32^ A total of 2240 diabetic individuals and 3483 controls were aggregated. The mean difference between the threshold of the diabetic group and the control group was found to be 3.19 dB (95% CI: 1.08‐5.19) higher in the diabetic group (1, 2). This difference is statistically significant with a *Z*‐score of 2.97, a *P*‐value of .003 and a *I* ^2^ of 89.0%. Despite the higher mean PTA thresholds observed in diabetics compared to controls, they remained within the range indicative of mild HL.

**Figure 3:**
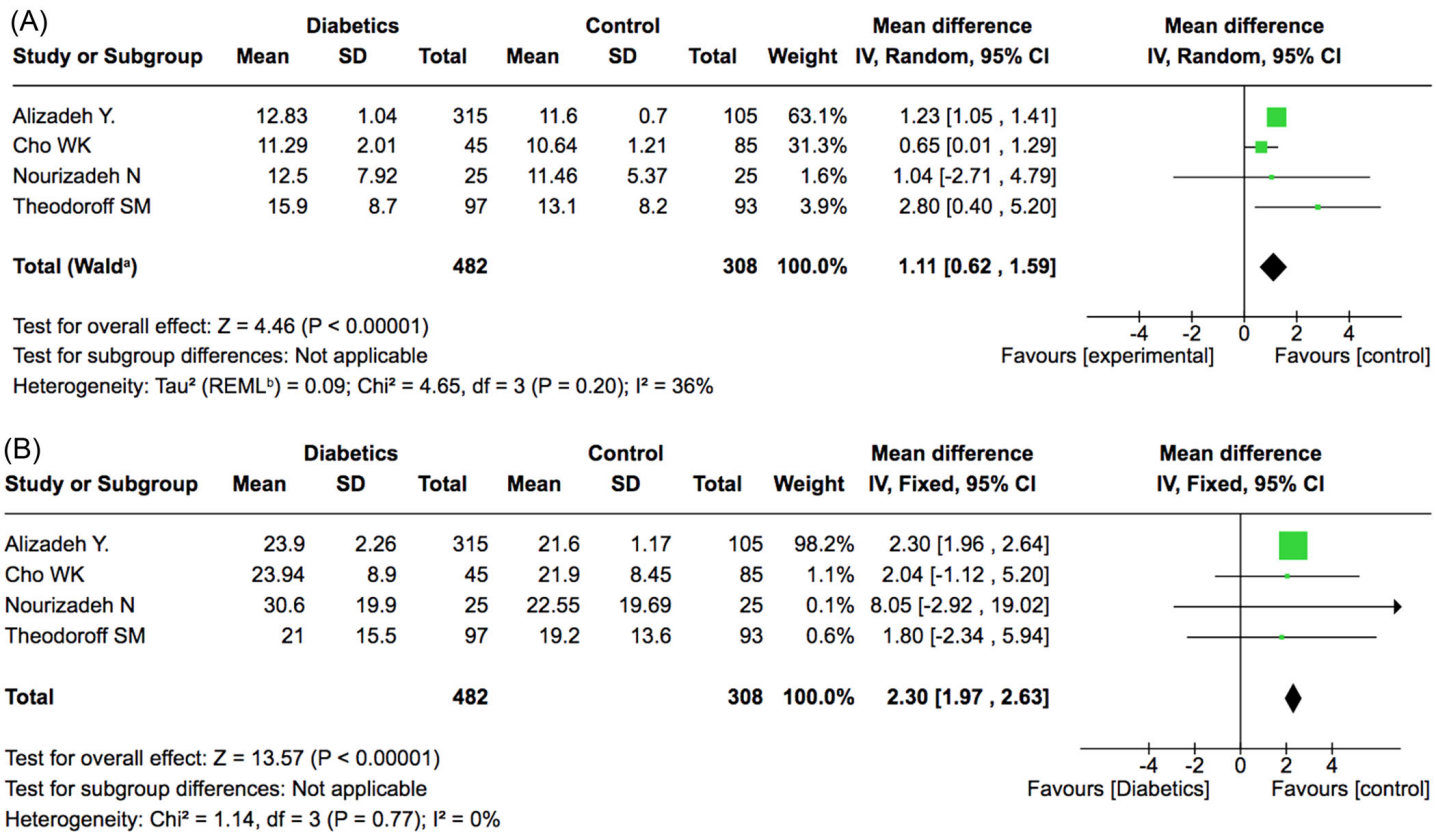
Pure tone thresholds for hearing low (500‐1000‐2000 Hz) (A) and high (4000‐8000 Hz) (B) frequencies.

Four studies provided data on mean PTA thresholds for low (500‐1000‐2000 Hz) and high‐ (4000‐8000 Hz) frequencies hearing.^16^, ^26^, ^28^, ^30^ A total of 484 people with diabetes and 308 controls were included in the analysis. Both analyses were statistically significant, but the difference in threshold was more pronounced in high frequencies (2.3 dB, 95% CI: 1.97‐2.63) (Figure 3B) than in low frequencies (1.11 dB, 95% CI: 0.62‐1.57) (Figure 3A). The values of hearing loss observed when analyzing high and low frequencies separately are lower than those in the overall analysis. This can be explained by the fact that different studies were used in each analysis. For the overall tone average analysis, 5 studies^16^, ^26^, ^28^, ^29^, ^32^ were included, but only three^16^, ^26^, ^28^ of them provided data on high and low frequencies. The high/low‐frequency meta‐analysis included those 3 studies, plus 1 additional study^30^ that reported data on high and low frequencies but did not provide overall tone average results.

### HbA1c Levels and Hearing Loss

**Figure 4:**
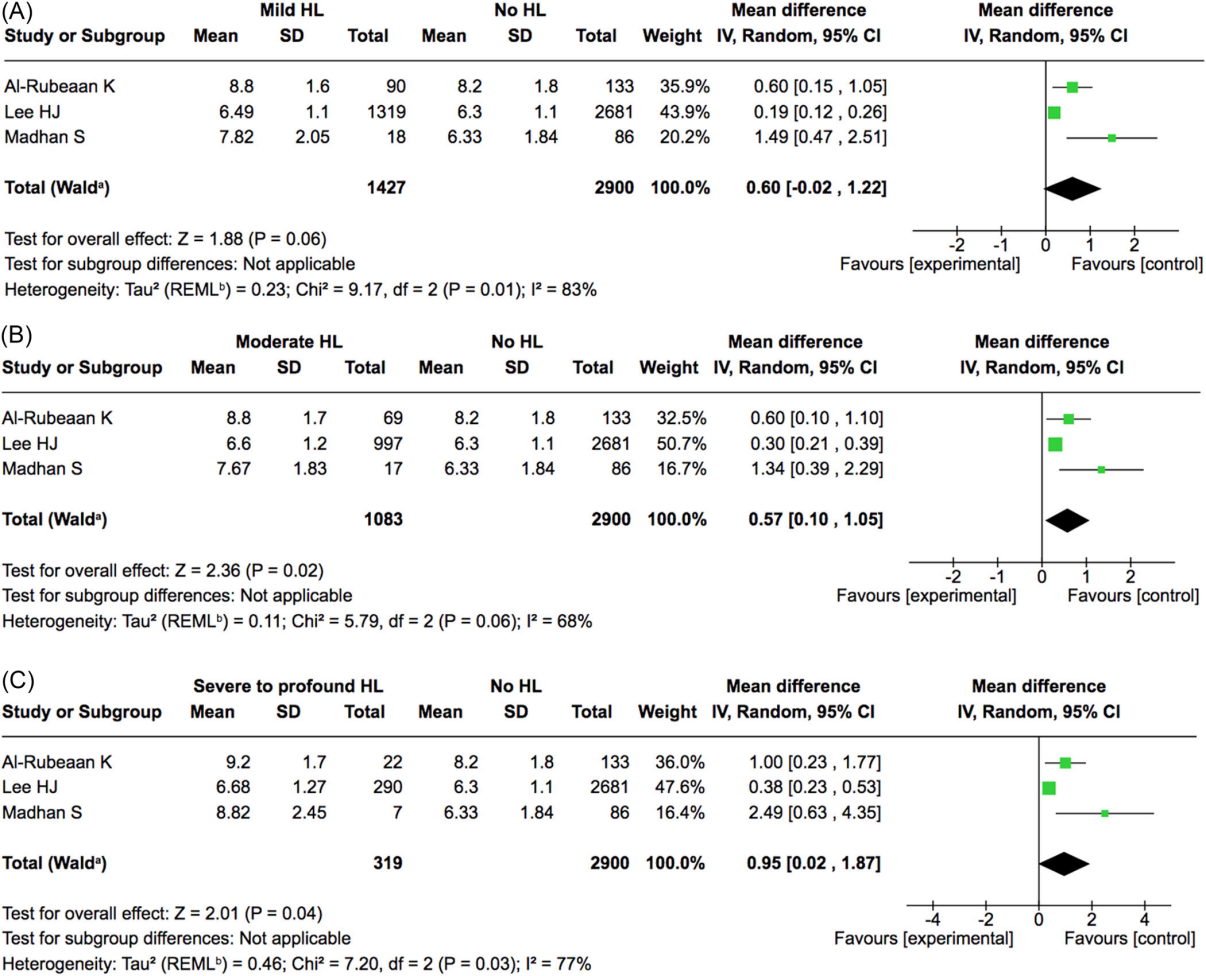
Relationship between HbA1c levels and hearing loss severity, categorised as mild (A), moderate (B), and severe‐to‐profound (C).

Three studies examined the relationship between HbA1c levels and HL severity, categorised as mild, moderate, and severe‐to‐profound HL. For mild HL, 1427 patients with HL and 2900 controls showed no significant difference in HbA1c levels (mean difference: 0.6%, 95% CI: −0.02‐1.22) (Figure 4A). In moderate HL, 1083 patients with HL showed a significant higher level of HbA1c than 2900 controls (0.57%, 95% CI: 0.1‐1.05) (Figure 4B). For severe to profound HL, 319 patients with HL showed a significant higher level of HbA1c than 2900 controls (0.95%, 95% CI: 0.02‐1.87) (Figure 4C).

## Discussion

The findings of this meta‐analysis indicate that individuals with T2DM have a 4.19 times higher risk of HL compared to controls. This result is more pronounced than that reported in other studies. For example, Horikawa et al^33^ observed that diabetic patients had 2.15 times higher risk compared to those without diabetes. Similarly, Akinpelu et al, reported 1.91 times higher risk of HL among patients with T2DM.^34^ Despite the discrepancy in the observed OR values across studies, this analysis substantiates the association between DM and HL.

The present analysis confirms that the percentage of patients with HL is higher in the diabetic group, however, further clarification is required. Most studies have established a threshold for detecting HL of greater than 25 dB, categorising all patients above this threshold as having HL, regardless of whether it is mild, moderate, or severe to profound. In these studies, most patients with HL have thresholds below 30 dB, indicating mild HL. These levels of HL may have little impact, so it is difficult to know from these results the true intensity of T2DM impairment and its impact on quality of life. Nor do they allow us to know whether those with mild impairment will go on to develop moderate or severe HL.

The results demonstrated a consistent elevation in auditory thresholds across all frequencies in individuals with diabetes compared to nondiabetic controls.^16^, ^25^, ^26^, ^28^, ^29^, ^30^, ^31^ The PTA analysis revealed a mean difference ranging from 1.08 to 5.29 dB, which is considered to be clinically insignificant. The reduction in sound perception was most pronounced at higher frequencies, between 4000 and 8000 Hz. These findings are corroborated by previous studies that have also reported elevated thresholds at higher frequencies.^35^, ^36^, ^37^, ^38^ Furthermore, Li et al employed additional methods beyond PTA results, namely extended high‐frequency audiometry (HFA), to investigate potential differences at higher frequencies. A comparison was made between a population of diabetics and a control group of healthy individuals. Even though all participants exhibited normal hearing thresholds according to the conventional PTA, those with diabetes displayed significantly elevated hearing thresholds at frequencies of 9 to 16 kHz in comparison to the control group, thereby indicating the presence of high‐frequency hearing impairments. Considering these findings, the authors concluded that HL in diabetic patients initially manifests at high frequencies and can be detected by HFA.^8^

The results of our analysis indicated a direct correlation between the duration of diabetes and the prevalence of HL. However, it is important to emphasize that the age of the patients can act as a confounding factor, as patients with longer duration of the DM are also older. Studies supporting this association with age include Al‐Abed et al^18^ and Madhan et al.^32^

Al‐Abed et al reported the highest mean age of patients and the highest prevalence of HL.^18^ Madhan et al had the youngest mean age of all the studies reviewed and the lowest prevalence of HL.^32^ However, other studies do not show this correlation between age and prevalence of HL in diabetic patients, for example, Al‐Rubeaan et al^21^ and Mishra et al,^17^ both with low mean age of diabetic cohorts and high prevalence of HL. These results could be explained by the average duration of the disease in these cohorts. Al Rubeaan et al cohort of diabetic patients had an elevated duration of diagnosis of DM.^21^ In our analyses, individuals with diabetes duration of 10 years or more, confront a 2.07 times greater risk of HL compared to those with a diabetes duration of less than 10 years. In the study by Mishra et al, this relationship cannot be verified as it does not provide data on the duration of DM.^17^ Other articles in the literature, which did not meet the criteria for inclusion in the meta‐analysis, also establish an association between age and HL, but do not take into account the duration of the disease.^39^, ^40^, ^41^

The available evidence indicates that the impact of T2DM on HL prevalence is more pronounced in younger individuals than in older cohorts, which may appear to be an unexpected finding.^34^, ^42^ Nevertheless, this observation is consistent with the view that diabetes duration and age are confounding variables. It is possible that aging may predispose the control group to a higher prevalence of HL, thereby reducing the differences between diabetic and control patients in older cohorts.^43^ Age and duration of diabetes must be considered in any study due to their strong relationship.

Pathological similarities have been identified in the cochlea during both the aging process and in individuals with DM,^3^ particularly at the basal portion of the spiral canal and the basement membrane within the capillaries of the stria vascularis. These observations may also explain the specific loss of hearing at higher frequencies.^6^, ^44^ Prior research has indicated that the diabetic condition exacerbates identifiable arteriosclerotic changes induced by aging.^45^ This suggests that diabetes may exacerbate the mechanisms underlying age‐related HL. Considering the existence of individual variations in susceptibility to age‐induced HL, it is plausible that DM may act as a contributing factor in the acceleration of the likelihood of developing age‐related HL. However, distinguishing between age‐related HL and hearing effects associated with DM in elderly diabetics represents a significant challenge. The 2 conditions have a common pathway, indicating that their effects may be additive.

Recent research supports that HL in diabetic patients may be related to subclinical microvascular disease, particularly through the role of microangiopathy, including retinopathy, nephropathy, and neuropathy. Emerging research suggests a potential link between these microvascular complications and hearing impairment in diabetic adults.^46^ Chronic hyperglycemia can damage small blood vessels in the inner ear, leading to reduced oxygen and nutrient supply.^47^ This results in vascular changes, such as thickened capillary walls and decreased blood flow, which may cause degeneration of the stria vascularis and damage to outer hair cells, both essential for sound transduction.^48^ This may explain why higher HbA1c levels, indicative of poorer disease control, are observed in individuals with severe to profound HL, with a difference approaching 1 full percentage point in our results.

Finally, the analysis of the combined data from 6 studies^19^, ^20^, ^21^, ^22^, ^23^, ^29^ indicates that gender does not appear to influence the prevalence of HL. However, the results suggest a slight inclination towards male subjects, which is consistent with findings from other researchers.^9^, ^49^, ^50^, ^51^, ^52^ This trend could be attributed to males traditionally being more exposed to risk factors such as tobacco use, hypertension, alcohol consumption, or occupations involving noise exposure.

### Limitations of the Study

This meta‐analysis is subject to a number of limitations that may give rise to bias. First, the exclusion of publications in languages other than English and Spanish may have resulted in the omission of studies that could have provided valuable findings. Second, focusing the analysis on the PTA as a hearing test may result in a narrower range of findings in comparison to other tests, such as ABR.

The results of the included studies demonstrated considerable heterogeneity. This heterogeneity can be attributed to several factors, including the characteristics of the diabetic cohorts, such as the mean age and duration of the illness, differences in the study populations, variations in the proportion of diabetic to control participants, and discrepancies in the prevalence of HL among control subjects across the included studies.

The establishment of a causal relationship between T2DM and HL remains challenging due to the lack of knowledge regarding the hearing levels of individuals prior to the onset of DM. Prospective studies are required to investigate the progressive changes in hearing function in diabetics and matched controls, with the potential to elucidate a causal relationship. Diabetics exhibit variability in various aspects, including treatment modalities and glycemic control levels, which may influence hearing thresholds. The randomization of patients to treatment groups in prospective studies could assist in the assessment of the effects of these covariates.

Cardiovascular comorbidities were not assessed. Recent studies have found a complex association between cardiovascular disease and sensorineural hearing loss, in particular an association with coronary heart disease and coronary artery bypass grafting, but not with hypertension or hyperlipidaemia.^53^ Given the high prevalence of cardiovascular disease in diabetic patients, these factors may act as confounders and limit the interpretation of our results.

Ultimately, the studies encompass disparate population cohorts, the majority of which are age‐matched. However, the influence of environmental factors on the exacerbation of diabetic hearing impairment remains uncertain.

### Strengths of the Study

This review presents novel insights into the correlation between T2DM and HL, including the following: (1) the performance of combined data analysis to obtain summary measures of odds ratio estimates concerning the impact of T2DM on hearing; (2) the revelation of a higher prevalence of at least mild forms of HL among diabetics compared to controls; (3) the indication of PTA thresholds indicating a mild degree of HL, especially at higher frequencies; (4) the analysis of data related to diabetes duration and its correlation with HL prevalence; (5) the investigation of the association between gender and HL; and (6) the analysis of HbA1c levels on HL thresholds.

### Future Implications in the Clinical Setting

In accordance with the established guidelines, pharmacological treatment is recommended upon the identification of microvascular disease, with the objective of delaying its progression.^54^ Given that HL may emerge as a consequence of microangiopathy, a worsening of pre‐existing HL, the new onset of HL or the presence of other microvascular disease in diabetic patients could prompt the initiation or intensification of diabetes management to prevent further deterioration of auditory function. Recent research indicates that effective glycemic control reduces the incidence of HL and slows the progression of HL among individuals with T2DM.^55^, ^56^ However, it is also important to note that the mean decibel difference between diabetic and control groups may pose challenges for implementation of treatment plans in a clinical setting. Further research is warranted.

## Conclusion

The findings of this meta‐analysis suggest that the prevalence of hearing loss among individuals with diabetes ranges from 40.6% to 71.9%. The risk of hearing loss is 4.19 times higher in this group compared to the control group. This risk is predominantly observed at higher frequencies. Furthermore, HbA1c levels appears to be correlated with the severity of hearing loss. Finally, the duration of diabetes appears to be a significant factor in the prevalence of hearing loss.

Hearing loss in DM may be a consequence of subclinical microvascular disease. This fact could potentially serve as an early warning sign, suggesting that closer monitoring is necessary, as well as the adaptation of treatment plans to minimize the occurrence or progression of hearing loss.

## Author Contributions

All the authors have made substantial contributions following the rules of the International Committee of Medical Journal Editors (ICMJE). **Miguel Caballero‐Borrego**: reviewer of the articles and writer of the paper. **Ivan Andujar‐Lara**: reviewer of the articles and conduct of the meta‐analysis.

## Disclosure

### Competing interests

None.

### Funding source

None.

## Supporting information


**SUPLEMENTAL MATERIAL.** The PRISMA 2020 checklist has been submitted as a supplementary file to document adherence to systematic review reporting standards.

## Data Availability

This review includes articles available on PubMed and Scopus.
